# Invertebrate communities in springs across a gradient in thermal regimes

**DOI:** 10.1371/journal.pone.0264501

**Published:** 2022-05-05

**Authors:** Agnes-Katharina Kreiling, Daniel P. Govoni, Snæbjörn Pálsson, Jón S. Ólafsson, Bjarni K. Kristjánsson

**Affiliations:** 1 Department of Aquaculture and Fish Biology, Hólar University, Sauðárkrókur, Iceland; 2 Institute of Life and Environmental Sciences, University of Iceland, Reykjavík, Iceland; 3 Department of Terrestrial Zoology, Faroe Islands National Museum, Tórshavn, Faroe Islands; 4 Marine and Freshwater Research Institute, Hafnarfjörður, Iceland; University of Eldoret, KENYA

## Abstract

In many respects, freshwater springs can be considered as unique ecosystems on the fringe of aquatic habitats. This integrates their uniqueness in terms of stability of environmental metrics. The main objective of our study was to evaluate how environmental variables may shape invertebrate diversity and community composition in different freshwater spring types and habitats within. In order to do so, we sampled invertebrates from 49 springs in Iceland, where we included both limnocrene and rheocrene springs. At each site, samples were taken from the benthic substrate of the spring (“surface”) and the upwelling groundwater at the spring source (“source”). To collect invertebrates from the spring sources we used a modified method of “electrobugging” and Surber sampler for collecting invertebrates from the surface. In total, 54 invertebrate taxa were identified, mostly Chironomidae (Diptera). Chironomid larvae also dominated in terms of abundance (67%), followed by Ostracoda (12%) and Copepoda (9%). The species composition in the surface samples differed considerably between rheocrene and limnocrene springs and was characterised by several indicator species. Alpha diversity was greater at the surface of springs than at the source, but the beta diversity was higher at the source. Diversity, as summarized by taxa richness and Shannon diversity, was negatively correlated with temperature at the surface. At the source, on the other hand, Shannon diversity increased with temperature. The community assembly in springs appears to be greatly affected by water temperature, with the source community of hot springs being more niche-assembled (i.e., affected by mechanisms of tolerance and adaptation) than the source community of cold springs, which is more dispersal-assembled (i.e., by mechanisms of drift and colonization).

## Introduction

Invertebrate community assembly within freshwater ecosystems is shaped by deterministic and stochastic processes [1,2]. When stochastic processes such as ecological drift and chance colonization are relatively more important than deterministic processes, the resulting communities can be characterized as dispersal-assembled [3], while deterministic processes based on individual species traits, such as tolerance and adaptation towards environmental attributes, lead to niche-assembled communities [4]. Niche-assembled communities show more predictable community composition, whereas dispersal-assembled communities result in a higher site-to-site variation (beta diversity) with otherwise similar environmental conditions [1].

Springs are known to be stable habitats with little temporal fluctuation in environmental variables such as temperature and chemical composition [5–7]. The great faunistic individuality and “uniqueness” in invertebrate community composition of freshwater springs has been emphasised by many researchers [8–12]. Although the patterns may vary depending on the dispersal abilities of invertebrates, e.g., between species with and without winged adults [12], there seem to be clear underlying trends of non-random patterns of species diversity and composition in springs, associated to environmental variables such as temperature [9,13], altitude [9,11], and spring type [14,15], as well as historical and geographical factors [16]. Community assembly in springs might differ depending on the dominant taxonomic group. Communities dominated by insects (temporal freshwater invertebrates) with a flying terrestrial adult stage may be more stochastically assembled in comparison to communities dominated by crustaceans (permanent freshwater invertebrates), whose entire life cycle is confined to aquatic habitats. Similarly, the proportion of crenobiont taxa (obligate spring fauna) in a community may influence the assembly processes.

Being a geologically young island, Iceland has a unique abundance of freshwater springs, emerging mostly at the edge of lava fields within or along the volcanically active zone which crosses the country from the southwest to the northeast. Due to the occurrence of geothermal areas, springs with temperatures above 14°C, classified as hot springs [17,18], are common. The high number of springs, expressing thermal stability at a wide range of temperatures, makes Iceland an excellent setting for a large natural experiment, allowing us to study how temperature may shape spring invertebrate communities, and to investigate possible mechanisms behind community assembly in spring habitats. An additional advantage is that the system is comparably simple, as the invertebrate fauna in general and the freshwater invertebrate fauna in particular consists of few species in Iceland [19]. Studies on geothermally heated streams in Southwest Iceland have revealed that macroinvertebrate and meiofaunal community structure change dramatically across a thermal gradient [20]. Furthermore, warming proved to simplify food-web structure and shorten the pathways of energy flux between consumers and resources [21], causing changes in community structure [22].

In addition to temperature, Icelandic springs vary in spring type, altitude, and other physical and chemical factors. Spring type (limnocrene—forming a pond, and rheocrene—forming a stream) influences hydraulic conditions and habitat structure and has been shown to affect the invertebrate community composition in springs, with limnocrene springs having a higher proportion of crustaceans in terms of abundance and number of species, than rheocrene springs [15]. Assuming that organisms colonize springs from the adjacent aquatic habitat, one would expect lentic taxa to dominate the fauna in limnocrene and lotic taxa in rheocrene springs [15].

Spring communities in Iceland have so far not been studied in relation to altitude, which is an important variable for spring communities in other areas of the world [9,11]. More than 75% of the land area of Iceland is higher than 200 m above sea level (asl) and over one third of the land area is above 600 m asl [23]. A high number of high-altitude springs can be found in Iceland, and the harsher environmental conditions in the highlands might shape the invertebrate community in highland springs.

Only few studies, e.g., [24,25] have focused on the biota within the sources of springs, at the point of groundwater emergence. Spring sources are often hard to access and sample with traditional methods, and this might be the reason why spring studies tend to neglect the source. In this paper we present a new sampling method based on electric fishing gear which allowed us to collect invertebrates from the spring source, i.e., from the ecotone between surface water and groundwater. In addition, we took samples from the eucrenal region, at the benthic substrate 2 m downstream of the source, which we refer to as “surface” habitat.

Here, our main objectives were to (I) test if invertebrate diversity and community composition were related to environmental variables, both at the spring source and at the surface, and (II) to examine which processes are dominating in shaping invertebrate community assembly in springs and whether such processes may differ among spring source and surface, taking into account different sampling methods. With respect to the first (I) objective, we predicted that (i) water temperature, spring type, and altitude would be important factors shaping the invertebrate community structure. We furthermore predicted (ii) a lower alpha diversity at the source due to a generally lower diversity in groundwater ecosystems [26] compared to surface waters. Considering our second (II) objective, we predicted that (iii) invertebrate communities in springs would be more influenced by stochastic processes resulting in high beta diversity when compared to deterministic processes. This prediction is based on the dominant taxa in Icelandic freshwaters being insects, especially Chironomidae (Diptera), which have generally good dispersal abilities. We also predicted that (iv) invertebrate communities at the source would show more influence of deterministic processes, reflected in a lower beta diversity, than at the surface. This is based on the source likely having a higher proportion of crenobiont taxa and taxa with limited dispersal abilities.

## Material and methods

**Fig 1:**
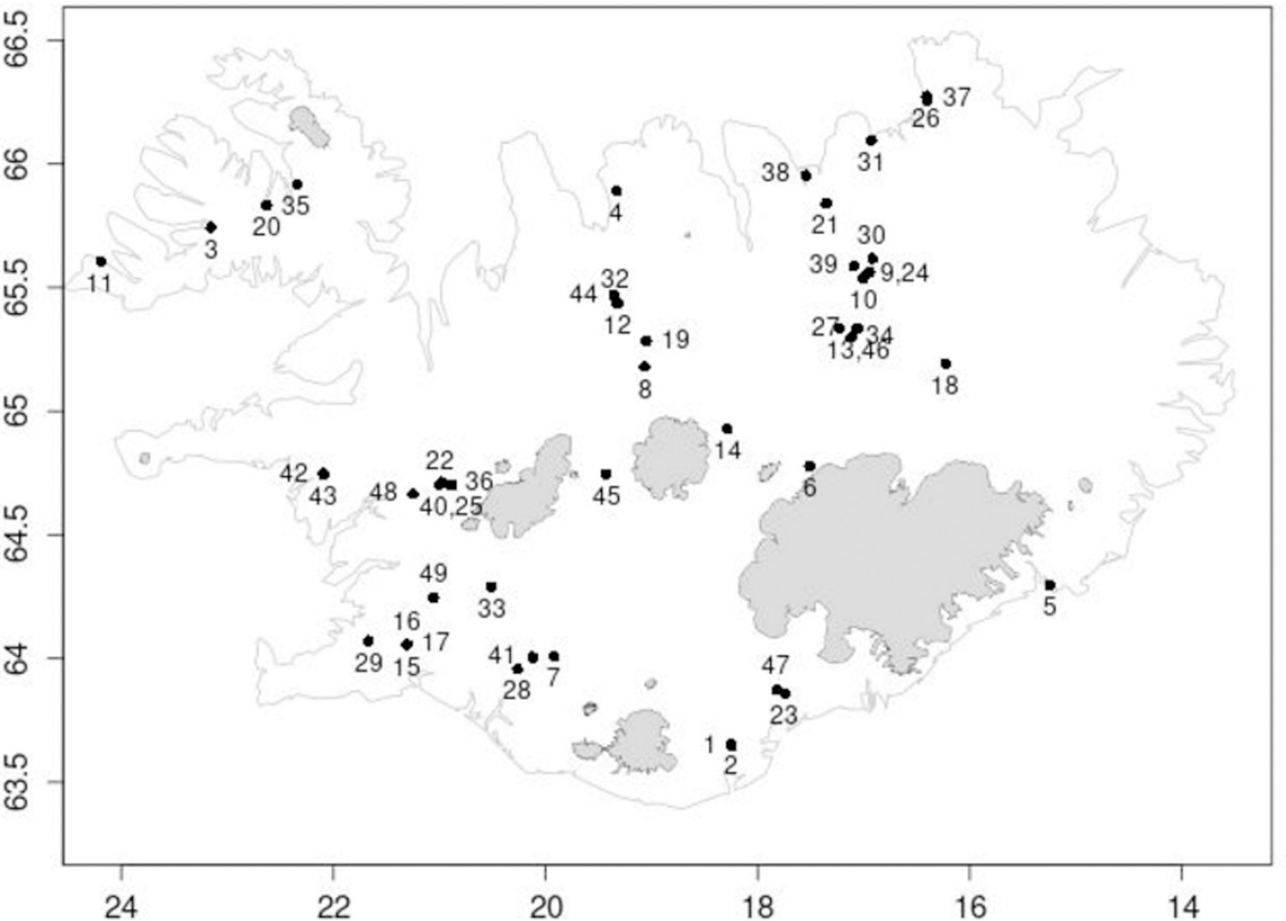
Location of the 49 freshwater springs in Iceland investigated in this study. Numbers refer to springs as listed in Table 2. The map was drawn using the map and mapdata libraries in R (https://cran.r-project.org/package=maps).

Invertebrate samples were collected from 49 springs in Iceland (Fig 1) between June and August in 2015 and 2016. Eighteen of the springs were limnocrene and 31 were rheocrene. Limnocrene springs were either located at the shore of shallow lakes or were discrete sources forming a small pond of still or slow-flowing water. The rheocrene springs all discharged into more or less fast-flowing streams and spring brooks, some of them just a few meters long. The springs were grouped into 25 cold (<5°C), 14 tepid (5–14°C) and 10 hot (>14°C at the source) springs, following the classification of [17] and [18]. Temperature, conductivity, oxygen saturation, and pH of each sampling location were measured at the time of sampling using a Hydrolab DS5 multi-probe sonde (Hach Hydromet, Loveland, CO, USA). In addition, HOBO temperature loggers (Onset Computer Corporation, Bourne, MA, USA) were placed at all the sites for one year to record the thermal regime. However, at some sites these loggers went missing, and data only exists for some of the sites. Furthermore, spring type, altitude, and presence of fish as top predators at the site were noted.

Two samples were collected at each spring site, using different sampling methods: one sample was collected directly from the upwelling groundwater at the source opening (referred to as “source”), using electrobugging [27] based on electric fishing gear. The method was modified specifically to sample invertebrates in spring sources and is described as follows: a drift net (30.5 x 45.7 cm) with a mesh size of 63 μm was removed from its frame to allow for complete flexibility of the opening of the net. Lead weights were tied to the grommets along the bottom of the drift net. The flexibility of the opening of the net allowed it to conform to the contours of the substrate, and the weights held the bottom in place. The top of the net was held upright by two stakes, which had the effect of keeping the net open. The net was placed closely in front of a source in order to catch the outflow. Modified electric fishing gear was used to apply electrical current to the spring. The copper plate serving as the cathode was put into the water body a few meters away from the source. For the anode, we replaced the commonly used pole with a flexible wire connected to an on/off switch on a box. The anode was inserted into the source duct as far as possible into the groundwater. Electricity (300 V, DC) was then applied for one minute. The electricity stuns the organisms within reach and causes them to detach from the substrate and flow into the driftnet. The second sample was collected at the benthic substrate approximately 2 m downstream of the source (referred to as “surface” to indicate the more surface water characteristics of the location as opposed to the groundwater nature of the “source” sample), using a 0.093 m^2^ Surber sampler (30.5 x 30.5 cm) with 63 μm mesh. This distance was chosen so that there would still be strong groundwater influence on the invertebrate communities, and that in limnocrene springs there would still be some water currents to carry invertebrates into the net. Surface samples were always collected prior to source samples. Most springs had only one source, and it was thus not possible to obtain more than one sample at a time, hence the lack of replicates. Samples were preserved in 70% ethanol.

Invertebrates were sorted under a dissecting microscope, counted and identified to the lowest possible taxonomic level. Amphipoda, Plecoptera, Coleoptera, Trichoptera, Chironomidae (Diptera), and Ephydridae (Diptera) were identified to species level. Ostracoda were identified to species level only for the limnocrene springs. Acarina were classified into Halacaridae, Hydrachnidia, and three different morphotypes of Oribatida (a, b, and c). Chironomid larvae were mounted on glass microscope slides and fixed in Hoyer’s mounting medium [28]. All chironomid larvae were processed unless their number per sample exceeded 250, in which case a random subsample of 200 individuals was identified. The total number for each taxon in the sample was then calculated based on its proportion in the subsample. Chironomid larvae were identified to lowest possible taxonomic level under a Leica DM4000B compound microscope (Leica Microsystems, Mannheim, Germany), using keys by [29] and [30]. Other invertebrate groups were identified using keys by [31] for Trichoptera larvae, [32] for Plecoptera nymphs, [33] for Ephydridae imagines, [34] for aquatic Coleoptera larvae and imagines, and [35] for Acarina. Ostracoda of the limnocrene springs were identified using the key by [36].

Statistical analysis was done using the software R (version 3.4.1) [37]. As a measure of alpha diversity, we calculated taxa richness (N_0_), Shannon diversity (N_1_), and Shannon evenness (as E = N_0_/N_1_; Hill’s ratio) according to [38], using the *diversity* function in the *vegan* package [39]. The dependence of Shannon diversity and evenness as response variables on the environmental variables, spring type, altitude, geographical position (latitude and longitude), and their interactions with the sampling location within the spring (source and surface) were analysed with multiple linear regressions. Taxa richness was analysed with generalized linear models (*glm* function in the *stats* package, [37]) with the Poisson link function. The distribution of the residuals was analysed to evaluate whether the assumptions of the tests were met. To assess the collinearity of the environmental variables, correlations between the environmental variables were calculated (function *chart*.*Correlation* in *PerformanceAnalytics* package, [40]), as well as the variance inflation factor (function *vif* in *car* package, [41]). In cases of high collinearity (VIF > 2), we retained the independent and most representative variables but excluded the others from subsequent tests. This resulted in initial models with the explanatory variables temperature, spring type, altitude, latitude, longitude, and their interactions with sampling location (source and surface). Initial models were simplified by a step-wise procedure, excluding the least significant variable until the minimal adequate model was reached. Model selection was based on the results of ANOVA tests, to test if the models significantly differed, and the Akaike information criterion (AIC).

The dependence of differences in community composition, summarized with Bray-Curtis distances (beta diversity), on the same explanatory variables as for the alpha diversity indices, were analysed by running a permutational multivariate analysis of variance (*adonis* function in the *vegan* package). The ordination of samples based on the beta diversity, and the association of the environmental variables to the main axes were visualised with nonmetric multidimensional scaling (NMDS) using the functions *metaMDS* and *envfit* in the *vegan* package. The beta diversity was further assessed by analysing the Sørensen dissimilarity, based solely on the presence or absence of taxa at each site, using the function *betadiver* in *vegan* with method = 1. To evaluate whether the degree of differentiation varied between the source and the surface samples, pairwise differences of the Sørensen dissimilarity among the source samples were compared with the dissimilarity among the surface samples with a Wilcoxon signed-rank test.

To explore preferences of taxa to spring type (rheocrene or limnocrene), we performed an indicator species analysis, as implemented in the functions *multipatt* and *signassoc* of the R package *indicspecies* [42]. The indicator value index is comprised of two components, specificity (A component) and fidelity (B component). Specificity indicates the positive predictive value of a species and is highest when a species is present in the target habitat group but not elsewhere. Fidelity indicates the sensitivity of a species as indicator and is highest when a species is present in all sites of the target habitat group [42].

## Results

The environmental conditions of the springs studied were quite variable, but were generally stable within springs (Table 1). Temperature, which was measured over the course of one year, was highly stable within the same spring with a standard deviation ranging from 0.04 to 1.23.

**Table 1:** Environmental characteristics of the springs studied.

Spring		Coordinates	Altitude [m asl]	Type	Fish presence	Location	Temperature [°C]	pH	Oxygen [%]	Conductivity [μS/cm¯^1^]
**1**	**Botnar I**	N 63°38.707’	36	rheocrene	yes	Source	5.7	8.11	72.5	113.7
		W 018°14.749’				Surface	5.61	8.03	74.2	115.9
**2**	**Botnar II**	N 63°39.275’	33	limnocrene	yes	Source	7.35	7.91	78.5	108.4
		W 018°15.142’				Surface	7.19	7.96	77.4	108.9
**3**	**Dynjandi**	N 65°44.604’	305	rheocrene	no	Source	2.31	7.63	47.6	50.9
		W 023°09.302’				Surface	2.81	7.64	48.1	50.9
**4**	**Enni**	N 65°53.371’	151	rheocrene	no	Source	4.65	7.44	76	81.6
		W 019°19.755’				Surface	4.45	7.27	75.9	80
**5**	**Friðsæld**	N 64°17.753’	27	rheocrene	no	Source	4.9	7.44	49.7	131.4
		W 015°14.764’				Surface	4.83	7.37	49.8	129.7
**6**	**Gæsavötn**	N 64°46.697’	928	rheocrene	no	Source	7.18	7.49	1.7	201
		W 017°30.687’				Surface	7.97	7.48	13.7	201
**7**	**Galtalækur**	N 64°00.453’	128	rheocrene	no	Source	5.4	7.99	73.1	151.8
		W 019°55.148’				Surface	5.09	7.93	72.3	151.8
**8**	**Goðdalafjall**	N 65°10.792’	570	rheocrene	no	Source	3.55	8.03	31.5	148.2
		W 019°03.972’				Surface	3.57	7.96	31.5	147.6
**9**	**Grænavatn Norður**	N 65°32.905‘	291	limnocrene	yes	Source	6.49	8.92	65.5	138.9
		W 016°58.908‘				Surface	6.49	8.93	60.6	139.5
**10**	**Grænavatn Suður**	N 65°32.205’	285	limnocrene	yes	Source	4.23	8.82	67.7	100.5
		W 017°00.477’				Surface	4.45	8.98	63.3	100.4
**11**	**Hænuvík**	N 65°36.420’	45	rheocrene	no	Source	5.02	7.37	46.3	106.3
		W 024°11.776’				Surface	5.76	7.24	46.5	102.3
**12**	**Hafgrímsstaðir**	N 65°26.184‘	82	rheocrene	no	Source	48.2	9.09	5.1	224
		W 019°19.205‘				Surface	43.19	9.21	3.9	248
**13**	**Hagalækur**	N 65°20.022’	441	rheocrene	no	Source	7.27	8.6	69.3	NA
		W 017°03.430’				Surface	5.52	9	70.5	NA
**14**	**Háöldur**	N 64°55.627’	767	rheocrene	no	Source	25.84	9.31	2.1	130.2
		W 018°16.947’				Surface	25.5	9.25	1.9	129.9
**15**	**Hengill IS6a**	N 64°03.426‘	406	rheocrene	no	Source	14.42	7.59	70.2	209
		W 021°18.244‘				Surface	11.49	7.43	63.7	214
**16**	**Hengill IS7**	N 64°03.407‘	384	rheocrene	no	Source	5.77	7.64	74.5	86
		W 021°18.391‘				Surface	5.38	8.19	74.8	86.9
**17**	**Hengill IS8**	N 64°03.414‘	381	rheocrene	no	Source	17.15	7.44	66.8	281
		W 021°18.439‘				Surface	16.59	7.54	66.1	287
**18**	**Herðubreiðarlindir**	N 65°11.548’	493	limnocrene	yes	Source	5.6	6.8	66.3	137.8
		W 016°13.508’				Surface	5.93	6.78	65.6	137.2
**19**	**Hofsvellir**	N 65°17.013’	354	rheocrene	no	Source	6.12	6.28	47.6	145.9
		W 019°02.850’				Surface	6.61	6.38	47.9	161.7
**20**	**Hörgshlíð**	N 65°49.885’	20	rheocrene	no	Source	32.43	8.35	39.1	13.4
		W 022°37.657’				Surface	32.23	9.05	58.1	190
**21**	**Hraun**	N 65°50.414’	34	rheocrene	yes	Source	5.21	7.46	70	156.2
		W 017°21.289’				Surface	4.8	7.52	65.4	156.3
**22**	**Hrauná**	N 64°42.261’	71	rheocrene	yes	Source	6.25	8.09	75	66.5
		W 020°59.870’				Surface	5.58	8.45	76	57.6
**23**	**Hruni**	N 63°51.547’	43	limnocrene	yes	Source	3.42	7.95	77.3	635
		W 017°44.486’				Surface	3.11	8.11	78.7	637
**24**	**Kálfaströnd**	N 65°33.759’	283	limnocrene	yes	Source	5.95	9.38	60	109.1
		W 016°56.710’				Surface	5.13	9.22	54	110.8
**25**	**Kiðárbotnar**	N 64°42.073’	128	limnocrene	yes	Source	3.39	9.43	78	46.4
		W 020°52.805’				Surface	3.12	9.47	77.7	46.4
**26**	**Klapparós**	N 66°16.481’	9	limnocrene	yes	Source	4.05	7.69	79	83
		W 016°24.530’				Surface	3.82	7.53	78.3	82.4
**27**	**Krákárbotnar**	N 65° 19.852’	430	rheocrene	no	Source	8.58	8.77	69.5	46.3
		W 017°04.654’				Surface	5.91	8.67	69.1	112
**28**	**Lækjarbotnar Hol**	N 63°57.422’	78	rheocrene	yes	Source	5.36	8.17	77.1	120
		W 020°15.892’				Surface	4.71	7.94	76.2	120
**29**	**Lækjarbotnar Rvk**	N 64°04.287’	121	rheocrene	no	Source	5.32	8.69	80.6	75.4
		W 021°40.107’				Surface	3.67	8.59	78.7	74.8
**30**	**Langivogur**	N 65°37.012’	286	limnocrene	yes	Source	19.79	8.34	75.1	434
		W 016°55.000’				Surface	13.41	8.37	62.4	300
**31**	**Lón**	N 66°05.785’	6	limnocrene	yes	Source	4.92	8.02	77.8	78.3
		W 016°55.514’				Surface	4.37	8.18	78	78.7
**32**	**Mælifellslaug**	N 65°26.557’	78	limnocrene	no	Source	23.44	8.72	3.9	182.3
		W 019°20.199’				Surface	21.59	7.69	3.2	192.8
**33**	**Miðhúsaskógur**	N 64°17.373’	184	limnocrene	no	Source	3.36	9.38	80.2	36.3
		W 020°30.706’				Surface	2.4	9.29	78.1	35.8
**34**	**Mótunga**	N 65°17.942’	437	rheocrene	no	Source	4.5	8.96	66.5	96.4
		W 017°07.114’				Surface	4.11	8.87	62.6	96.4
**35**	**Nauteyri**	N 65°55.039’	31	limnocrene	no	Source	25.5	8.66	27.4	162.7
		W 022°20.500’				Surface	29.77	8.93	41	167.5
**36**	**Oddar**	N 64°42.110’	124	limnocrene	yes	Source	3.71	9.41	78	46.4
		W 020°53.787’				Surface	3.11	9.38	77.6	46.4
**37**	**Presthólar**	N 66°15.477’	28	rheocrene	yes	Source	4.11	7.95	76.7	93.7
		W 016°24.081’				Surface	3.78	7.92	76.8	92.6
**38**	**Sandur**	N 65°57.223’	8	limnocrene	yes	Source	3.94	7.57	66.1	154.8
		W 017°32.701’				Surface	3.77	7.42	55	151.9
**39**	**Sikið**	N 65°35.210’	284	limnocrene	no	Source	5.05	7.7	51.5	156
		W 017°05.573’				Surface	4.67	7.65	44.6	155.7
**40**	**Sílatjörn**	N 64°42.810’	100	rheocrene	yes	Source	3.99	7.6	75.3	57.1
		W 020°58.600’				Surface	3.85	7.72	75.3	56.6
**41**	**Skarðslækur**	N 64°00.306’	103	rheocrene	yes	Source	5.2	6.7	46.8	112.7
		W 020°07.110’				Surface	5.24	6.92	47.2	112.9
**42**	**Staðarhraun Bær**	N 64°44.610’	62	rheocrene	yes	Source	5.4	5.26	78.9	68.2
		W 022°05.647’				Surface	3.98	5.68	79.7	69.9
**43**	**Staðarhraun Kirkja**	N 64°44.855’	62	rheocrene	yes	Source	5.21	5.31	81.3	71.8
		W 022°05.812’				Surface	4.57	5.26	79.2	69.4
**44**	**Steinsstaðir**	N 65°28.162’	62	rheocrene	no	Source	42.62	8.48	80.1	265
		W 019°21.390’				Surface	40.24	8.47	86.3	266
**45**	**Svartárbotnar**	N 64°44.768’	568	rheocrene	yes	Source	3.24	9.75	37	86.9
		W 019°25.801’				Surface	3.3	9.83	39.2	86.9
**46**	**Svartárkot**	N 65°20.153’	382	rheocrene	no	Source	3.8	8.9	71.5	83.8
		W 017°13.926’				Surface	3.66	8.96	70.8	83.9
**47**	**Þverá**	N 63°52.396’	53	limnocrene	yes	Source	5.11	7.45	76.1	66.3
		W 017°49.199’				Surface	4.76	7.51	75.2	65.7
**48**	**Úlfsstaðir**	N 64°39.911’	56	rheocrene	no	Source	26.24	10.03	43	0.3
		W 021°14.963’				Surface	42.96	9.82	2.9	282
**49**	**Vatnsvik**	N 64°14.757’	109	limnocrene	yes	Source	3.71	8.89	74.6	53.1
		W 021°03.304’				Surface	3.64	8.71	76.1	52.5

The environmental variables were measured at the time of sampling, both at the source and the downstream benthic substrate (i.e., surface) of the investigated springs.

In total, 54 aquatic invertebrate taxa were identified (Table 2), the majority (51) of them belonging to the phylum Arthropoda. The most abundant and prevalent invertebrate groups were Chironomidae (Insecta, Diptera) (67%), followed by Ostracoda (Crustacea) (12%), and Copepoda (Crustacea) (9%). Chironomidae larvae was the dominant group both in terms of abundance and in number of taxa (26). As ostracoda were only identified to species in the limnocrene springs they were combined under the higher taxonomic level Ostracoda for analysis. Thus, of the 54 collected taxa, only 47 were included in the data analysis (Table 2) and discussed here.

**Table 2:** Invertebrates found in freshwater springs in Iceland.

Taxon	Acrn.	Surface samples		Source samples	
		rheocrene	limnocrene	rheocrene	limnocrene
GASTROPODA*	GAS	637	70	2	9
OLIGOCHAETA*	OLI	1512	1437	56	65
TARDIGRADA*	TAR	697	7976	4	8
ACARI	ACA				
Halacaridae*	Hal	12	5	13	7
Hydrachnidia*	Hyd	65	36	5	12
Oribatida a,b*	Ora	74	48	19	14
Oribatida c*	Orc	104	0	11	0
CLADOCERA*	CLA	8	258	0	41
COPEPODA*	COP	6804	1841	1712	834
OSTRACODA*	OST	10085	997	3908	97
*Bradleystrandesia affinis* (Fischer, 1851)		0	1	0	0
*Candona candida* (Müller, 1776)		11	53	0	0
*Cryptocandona reducta* (Alm, 1914)		1	0	0	0
*Cytherissa lacustris* (Sars, 1863)		0	4	0	0
*Limnocythere inopinata* (Baird, 1843)		0	3	0	0
*Potamocypris fulva* (Brady, 1868)		82	110	0	2
*Potamocypris pallida Alm*, *1914*		0	72	2	5
*Potamocypris villosa* (Jurine, 1820)		0	0	0	2
AMPHIPODA* Crangonyx islandicus Svavarsson & Kristjánsson, 2006	AMP	0	0	4	2
COLLEMBOLA*	COB	16	36	99	54
PLECOPTERA* Capnia vidua Klapálek, 1904	PLE	45	3	16	5
COLEOPTERA* Agabus bipustulatus (Linnaeus, 1767)	COL	4	1	5	0
TRICHOPTERA	TRI				
*Apatania zonella* (Zetterstedt, 1840)*	Azo	14	4	0	0
*Limnephilus affinis* Curtis, 1834*	Laf	5	0	0	0
*Limnephilus griseus* (Linnaeus, 1758)*	Lgr	19	1	3	3
*Limnephilus* sp.	Lph	0	1	0	0
CHIRONOMIDAE					
Podonominae					
*Parochlus kiefferi* (Garrett, 1925)*	Pki	58	3	1	0
Tanypodinae					
*Arctopelopia* sp. (*A*. *griseipennis* (van der Wulp, 1858))*	Arc	0	12	0	10
*Macropelopia* sp.*	Mac	66	7	7	21
*Procladius* sp. (*P*. *islandicus* (Goetghebuer, 1931))*	Pro	5	38	0	22
Diamesinae					
*Diamesa* spp.*	Dia	22201	941	4382	3822
*Pseudodiamesa* sp.*	Pse	64	24	0	0
Orthocladiinae					
*Chaetocladius* spp.*	Cha	2671	33	343	30
*Coryoneura fittkaui* Schlee, 1968*	Cof	116	0	0	0
*Cricotopus sylvestris* (Fabricius, 1794)*	Crs	365	69	13	45
*Cricotopus tibialis* (Meigen, 1804)*	Crt	5	80	0	56
*Cricotopus* sp.*	Cri	58	3	0	39
*Eukiefferiella claripennis* (Lundbeck, 1898)*	Euc	7	0	13	34
*Eukiefferiella minor* (Edwards, 1929)*	Eum	13067	586	1073	922
*Heterotrissocladius* sp. (*H*. *grimshawi* (Edwards, 1929))*	Het	0	16	0	0
*Limnophyes* sp.*	Lim	124	12	16	0
*Metriocnemus eurynotus* (Holmgren, 1883)*	Meu	236	0	16	18
*Metriocnemus fuscipes* (Meigen, 1818)*	Mfu	58	0	0	0
*Metriocnemus sp*.*	Met	0	0	6	7
*Orthocladius frigidus* (Zetterstedt, 1838)*	Ofr	6917	158	1597	1427
*Orthocladius oblidens* (Walker, 1856)*	Oob	959	218	5	327
*Orthocladius* sp.*	Ort	3252	468	1942	1437
*Paralimnophyes* sp.*	Par	3	0	0	0
*Rheocricotopus effusus* (Walker, 1856)*	Ref	230	7	1	0
*Smittia* sp.*	Smi	0	0	10	0
*Thienemanniella* sp. (*T*. *clavicornis* (Kieffer, 1911))*	Thi	3069	60	97	67
Chironominae					
*Chironomus* sp.*	Chi	0	2	0	18
*Micropsectra* sp.*	Mic	4398	239	258	371
SIMULIIDAE*	SIM	13	1	4	0
EPHYDRIDAE* Scatella tenuicosta Collin, 1930	EPH	479	0	20	1
DIPTERA other*	DIP	120	9	13	20
NEMATODA/NEMATOMORPHA*	NEM	336	85	17	10

Numbers represent total number of individuals found in rheocrene and limnocrene springs at the source and the surface. Taxa which were included in the data analysis are marked with an asterisk (*). The taxa acronyms (“Acrn.”) refer to the ones used in Figs 3 and 4.

**Fig 2:**
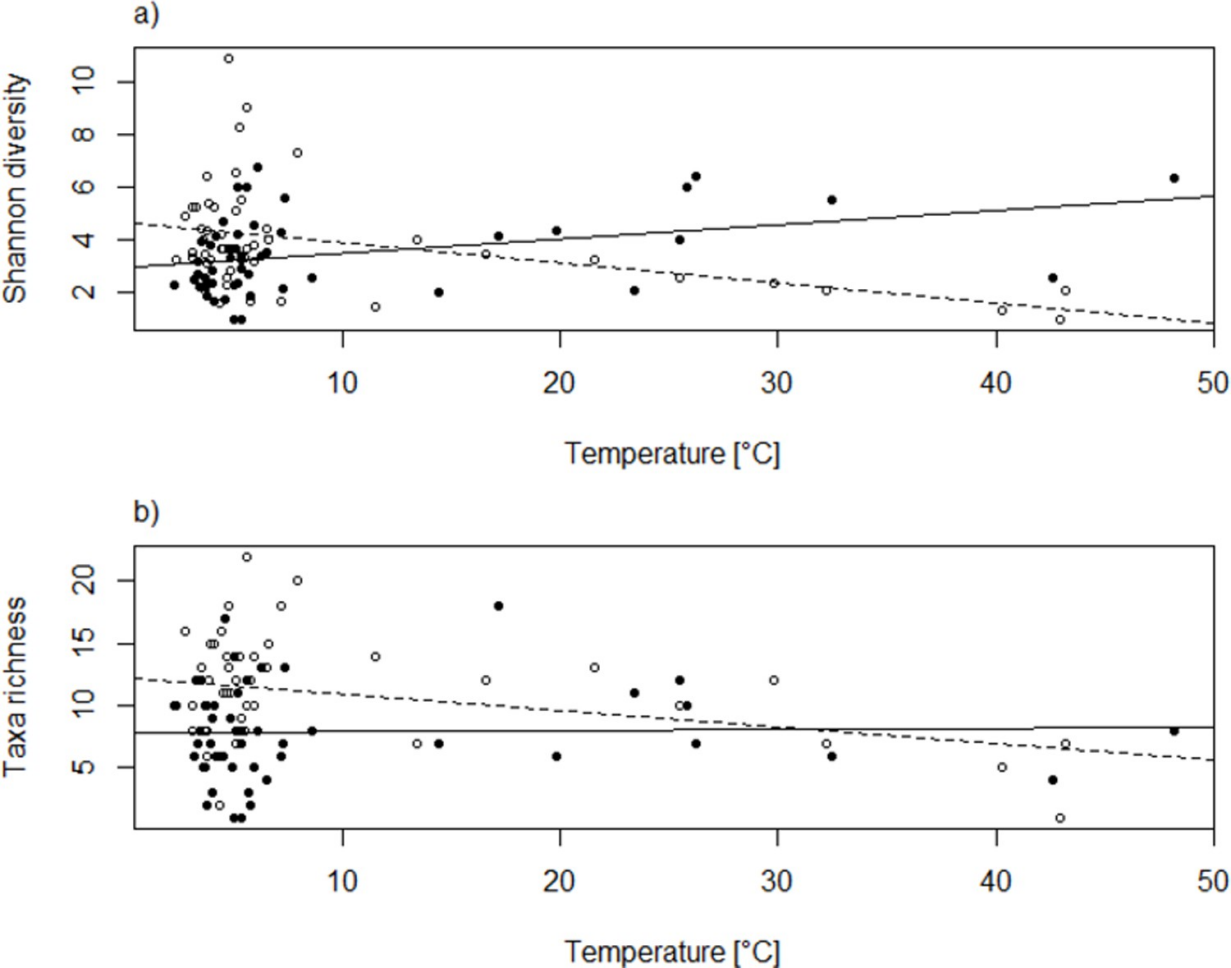
a-b. Diversity of invertebrates in Icelandic springs with respect to temperature. Shannon diversity (a) and Taxa richness (b) are shown in relation to spring temperature at the source (black dots) and the surface (white dots). Regression lines for the source samples are shown as continuous line and for the surface samples as broken line. The regression analysis is summarized in Table 5.

The different alpha diversity indices varied among the springs, with number of taxa ranging from 1 to 18 at the source and from 1 to 22 at the surface. Both taxa richness and Shannon diversity (S1 Table) were on average lower at the source than at the surface, with an average of taxa richness (± standard deviation) of 8 ± 3.8 and 11 ± 4.3, respectively, and an average Shannon diversity of 3.5 ± 1.48 and 4.0 ± 2.00. Mean evenness, on the other hand, was slightly higher at the source (0.5 ± 0.27) than at the surface (0.4 ± 0.21). Diversity indices were similar for rheocrene and limnocrene springs. Taxa richness was negatively correlated with temperature at the surface but was not correlated with temperature at the source (Fig 2B, Tables 3A and 4). Similarly, Shannon diversity decreased with increasing temperature at the surface, but increased at the source (Fig 2A, Table 3B). Furthermore, taxa richness was influenced by geographical position of the spring, and increased slightly towards the west of Iceland (Table 3A). Evenness was not influenced by environmental variables or sampling location but decreased westwards (Table 3C).

**Table 3:** Dependence of alpha diversity indices on environmental variables and sampling location within each spring (source or surface).

Diversity index	Variable	b	SE	t-value	p-value
**a)** Taxa richness					
	Temperature	-0.0003	0.005	-0.07	0.945
	Location	0.454	0.090	5.04	<0.001
	Longitude	0.037	0.015	2.44	0.015
	Temperature*Location	-0.015	0.007	-2.23	0.026
**b)** Shannon diversity					
	Temperature	0.054	0.023	2.40	0.018
	Location	1.747	0.443	3.94	<0.001
	Temperature*Location	-0.130	0.031	-4.15	<0.001
**c)** Shannon evenness					
	Temperature	0.006	0.003	1.79	0.077
	Location	-0.075	0.064	-1.17	0.247
	Longitude	-0.028	0.011	-2.49	0.015
	Temperature*Location	-0.005	0.005	-1.18	0.240

Slope (b), standard error (SE), t-values, and p-values are shown. a) Taxa richness. R^2^ = 0.199. b) Shannon diversity. R^2^ = 0.178. c) Shannon evenness. R^2^ = 0.143.

**Table 4:** Correlation matrix of some environmental variables from Icelandic springs.

Source	**Altitude**	-0.09	0.14	-0.38**	-0.02	Surface
	-0.07	**Temperature**	0.31*	-0.53***	0.42**	
	0.13	0.27	**pH**	-0.18	0.01	
	-0.41**	-0.48***	-0.18	**Oxygen**	-0.24	
	0.05	0.23	-0.10	-0.07	**Conductivity**	

The matrix above the variables shows correlations at the surface and below the variables at the source. Significant correlations are indicated by asterisks as *<0.05, **<0.01, ***<0.001.

**Fig 3:**
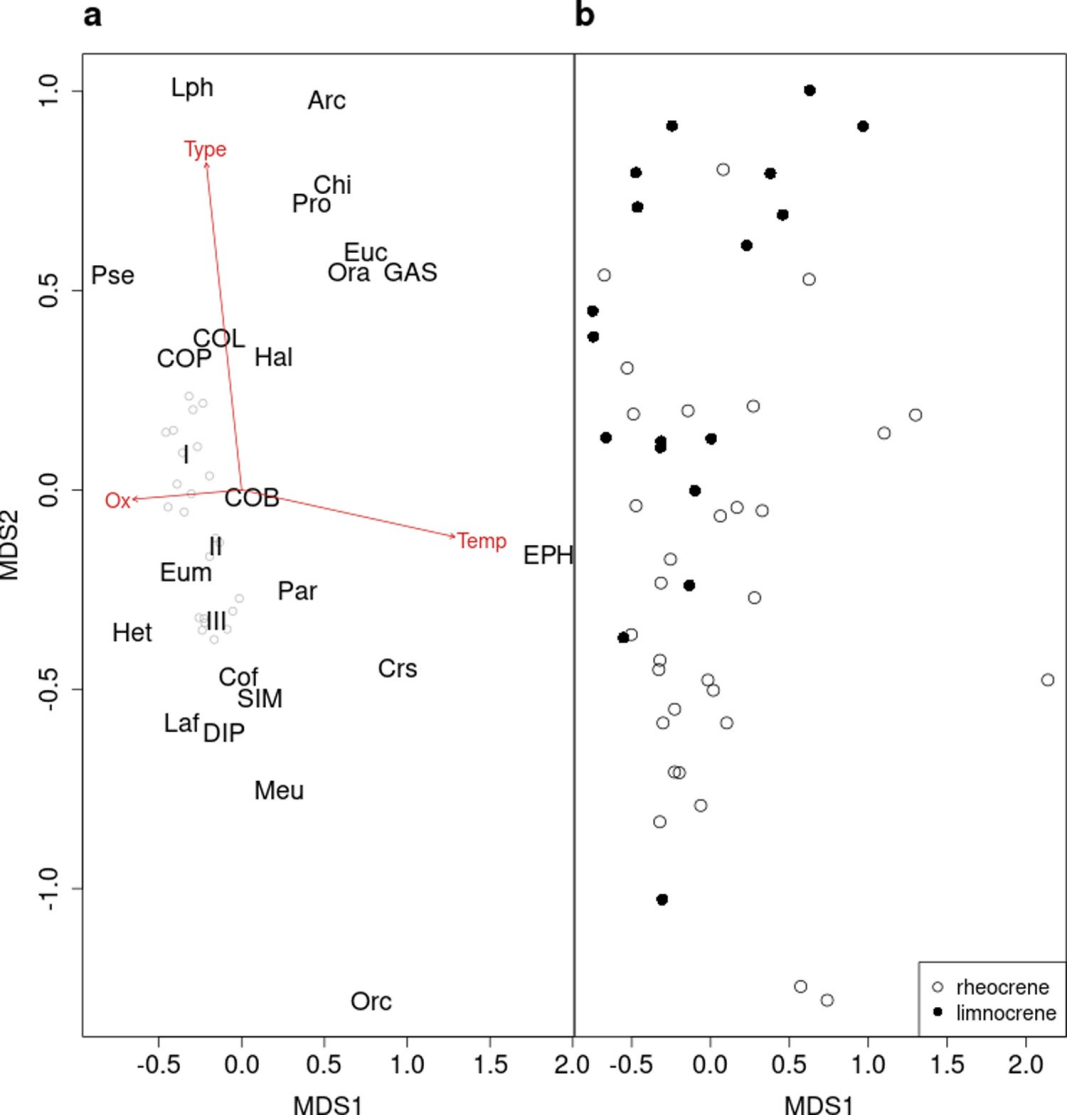
a-b. Non-metric multidimensional scaling of invertebrate taxa (a) and sites (b) for surface samples. Non-linear stress was 0.210. Significant associations (p < 0.05) of the environmental variables to the axes are shown with arrows (Ox = oxygen saturation, Type = spring type, Temp = temperature). Acronyms of invertebrate taxa (a) are listed in Table 2. Three clusters (I–III) of overlapping data points were defined for clarity reasons, containing the following taxa: Cluster I–Azo, CLA, Crt, Lim,Mac, Mic, NEM, OLI, Oob, OST, Ref, TAR; cluster II–Cha, Dia, Ofr; cluster III–Cri, Hyd, Lgr, Mfu, Ort, Pki, PLE, Thi. Spring sites (b) are classified as rheocrene and limnocrene springs.

**Fig 4:**
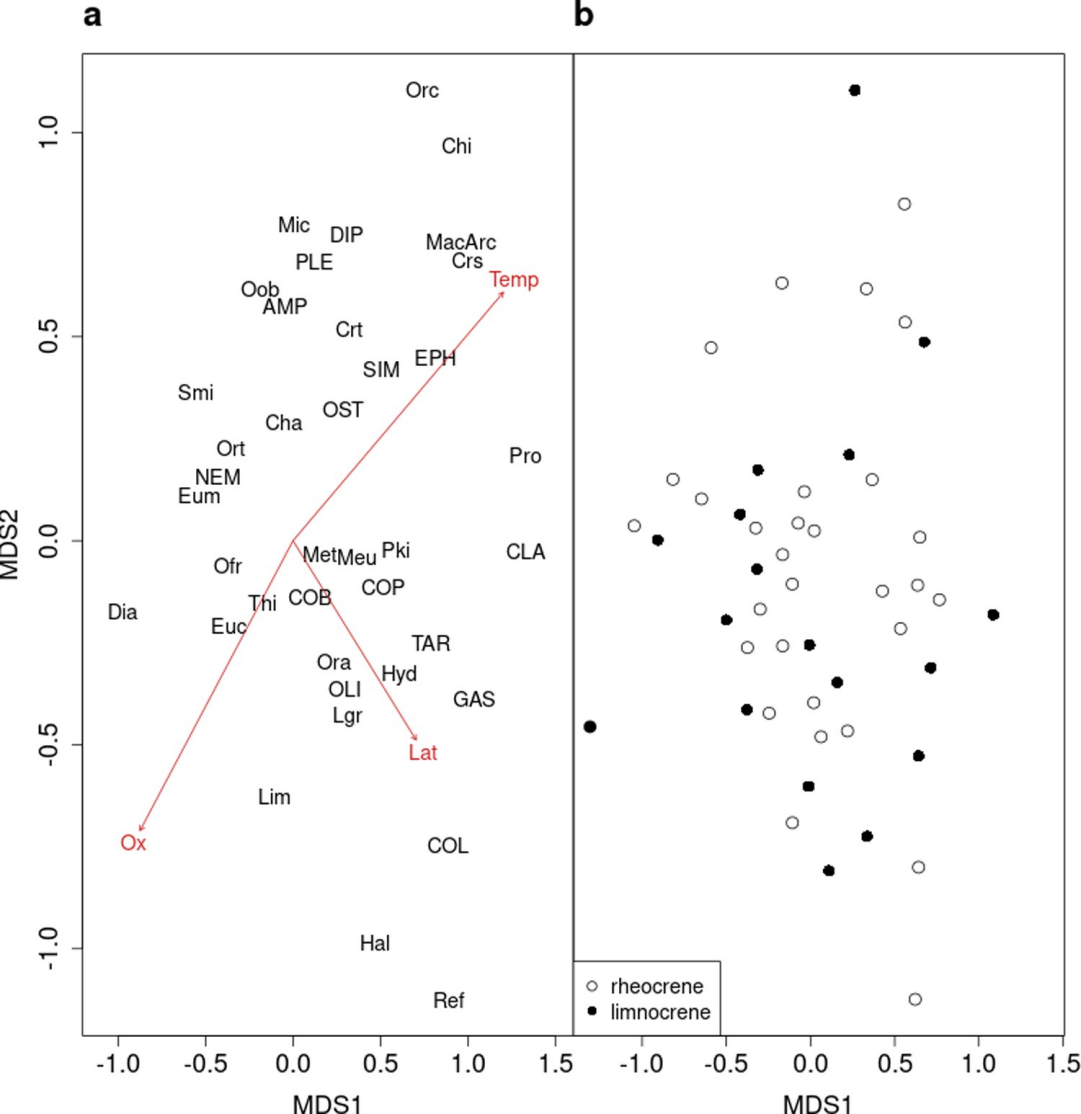
a-b. Non-metric multidimensional scaling of invertebrate taxa (a) and sites (b) for source samples. Non-linear stress was 0.18. Significant associations (p < 0.05) of the environmental variables to the axes are shown with arrows (Ox = oxygen saturation, Temp = temperature, Lat = Latitude). Acronyms of invertebrate taxa (a) are listed in Table 2. Spring sites (b) are classified as rheocrene and limnocrene springs.

Community composition was shaped by temperature, spring type, geographical position (latitude), and sampling location within the spring (Table 5). Less variation was observed among the surface samples (mean beta diversity = 0.547) than among the source samples (mean beta diversity = 0.605; V = 231830, p < 0.001). At both surface and source, the community composition changed with temperature and oxygen concentration (Figs 3A and 4A). Specifically, *Scatella tenuicosta* Collin (Ephydridae), *Cricotopus sylvestris* Fabricius (Orthocladiinae), Oribatida c, *Macropelopia* sp. (Tanypodinae), *Arctopelopia* sp. (Tanypodinae), *Procladius* sp. (Tanypodinae), *Chironomus* sp. (Chironominae), and Gastropoda were associated with high temperatures and low oxygen saturation. The chironomids *Orthocladius frigidus* Zetterstedt, *Thienemanniella* sp., *Diamesa* spp. (Diamesinae), *Cricotopus tibialis* Meigen, and *Eukiefferiella minor* Edwards (Orthocladiinae), as well as *Apatania zonella* Zetterstedt (Apataniidae, Trichoptera), Hydrachnidia, and *Capnia vidua* Klapálek (Plecoptera) were associated with colder parts of the temperature gradient. Community composition differed between rheocrenes and limnocrenes but was dependent on the location within springs as revealed by the interaction (Table 5); it differed at the surface (*adonis*, F Model = 2.78, p = 0.001, Fig 3B) but not at the source (*adonis*, F Model = 0.759, p = 0.737; Fig 4B). *Diamesa* spp., *E*. *minor*, *Micropsectra* sp. (Chironominae), *Orthocladius* spp., and Ostracoda were relatively more abundant in rheocrene, and Tardigrada and Cladocera in limnocrene springs (Table 2). In source samples, communities of rheocrene and limnocrene springs were more similar to each other, with *Diamesa* spp., *E*. *minor*, *Micropsectra* sp., *O*. *frigidus*, Ostracoda, and Copepoda as the dominant taxa (Table 2).

**Table 5:** Dependence of the invertebrate community composition on environmental variables and sampling location within each spring (source or surface).

Variable	F Model	r^2^	p-value
Location	3.654	0.033	0.001
Temperature	5.377	0.049	0.001
Type	2.311	0.021	0.005
Latitude	4.129	0.037	0.001
Longitude	1.476	0.134	0.082
Temperature*Location	1.776	0.016	0.021
Type*Location	1.737	0.016	0.030

Indicator species (Table 6) for rheocrene springs at the surface were *O*. *frigidus*, *Thienemanniella* sp., *Chaetocladius* sp. (all Orthocladiinae), as well as *S*. *tenuicosta* and not further identified Diptera larvae. On the other hand, *Arctopelopia* sp., *C*. *tibialis* (Orthocladiinae), and Cladocera were indicator species for limnocrene springs at the surface. No indicator taxa were identified for the source habitat.

**Table 6:** Indicator species of rheocrene and limnocrene springs at the surface.

Indicator species	Specificity (A component)	Fidelity (B component)	Indicator value index	p-value
**Rheocrene** springs:				
*O*. *frigidus*	0.962	0.742	0.85	0.007
Diptera larvae other	0.886	0.645	0.76	0.004
*Thienemanniella* sp.	0.967	0.581	0.75	0.015
*Chaetocladius* spp.	0.979	0.452	0.67	0.042
Ephydridae	1.000	0.290	0.54	0.039
Hydrachnidia	0.782	0.355	0.53	0.114
Plecoptera	0.897	0.290	0.51	0.099
*R*. *effusus*	0.950	0.161	0.39	0.432
*L*. *griseus*	0.917	0.129	0.34	0.372
Oribatida c	1.000	0.097	0.31	0.380
*M*. *eurynotus*	1.000	0.065	0.25	0.525
*M*. *fuscipes*	1.000	0.065	0.25	0.508
*C*. *fittkaui*	1.000	0.032	0.18	1.000
*E*. *claripennis*	1.000	0.032	0.18	1.000
*Paralimnophyes* sp.	1.000	0.032	0.18	1.000
*L*. *affinis*	1.000	0.032	0.18	1.000
**Limnocrene** springs:				
Cladocera	0.980	0.333	0.57	0.019
*Arctopelopia* sp.	1.000	0.222	0.47	0.014
*C*. *tibialis*	0.965	0.222	0.46	0.024
*Procladius* sp.	0.929	0.111	0.32	0.127
*Chironomus* sp.	1.000	0.056	0.24	0.387
*Heterotrissocladius* sp.	1.000	0.056	0.24	0.379

The components specificity and fidelity determining the indicator value are also represented (see further details in the methods section). P-values presents significant associations.

## Discussion

We predicted (i) that the main drivers of invertebrate diversity and community composition in Icelandic springs were water temperature, spring type, and altitude. The results of our study support this prediction partly, as invertebrate diversity was affected by temperature, but not by altitude or by spring type. The community composition of the surface samples, on the other hand, was affected by temperature and spring type, but not altitude. In addition, geographical position (as latitude) turned out to influence the diversity and community composition at the source. We furthermore predicted (ii) that the source would have a lower alpha diversity than the surface, and this prediction was supported by our results. Although we acknowledge possible differences that are the results of different sampling methods, there is a clear difference in specific indicator taxa occurrence which appears to be a result of environmental preference of the species. As the second objective of our study, we examined whether the processes of community assembly differed between spring source and surface, using beta diversity as a proxy to indicate the stochasticity of assembly. We had predicted (iii) that springs have a high beta diversity emphasising the relative importance of stochastic as opposed to deterministic factors during assembly. The results supported this prediction for cold springs, whereas hot spring communities seemed to be restricted by deterministic factors. Lastly, we predicted (iv) a lower beta diversity at the source than at the surface, but our results show the opposite.

Water temperature proved to be one of the most influential environmental variables for both invertebrate diversity and community composition. Invertebrate taxa were distributed along the temperature gradient in line with their reported ecology, e.g., [43]. So were, for example, the cold-stenotherm chironomids *O*. *frigidus*, *Thienemanniella* sp., and *Diamesa* spp. (Diamesinae) mainly found at the colder part of the temperature gradient, whereas *Arctopelopia* sp., *Procladius* sp., *C*. *sylvestris*, and *S*. *tenuicosta* were clearly associated with higher water temperatures. Myers & Resh [9] found a consistent core group of species occurring repeatedly in warm springs, whereas no such core group existed in cold springs. The core group of Icelandic hot springs seems to be comprised of the chironomid *C*. *sylvestris*, the ephydrid *S*. *tenuicosta*, Gastropoda, and the mite order Oribatida. The first three of those taxa were described as the “character animals of the absolutely hot springs” in Iceland by [17] (and see [18]). Although Tuxen listed one species of Oribatida, *Hydrozetes lacustris* Michael (Hydrozetidae), found in a 16°C warm spring [17], he did not specifically mention Oribatida in conjunction with hot springs. Our results, however, indicate that Oribatida are common in and characteristic for hot springs in Iceland and should thus be added to the core species group of hot springs.

The community composition of rheocrene and limnocrene springs was clearly different at the surface, but was more similar to each other at the source. Spring type was not reflected in taxa richness, Shannon diversity, and evenness, neither at the source nor at the surface. This is in accordance with a previous study on springs [15], which found that spring type affected community composition but not diversity. This underlines the importance of taking into account measures of both species diversity and species composition in ecological studies, especially in springs, as one might lose crucial information about habitat properties when focusing only on diversity indices.

The indicator species analysis revealed five taxa indicative for rheocrene springs at the surface, three of which, *O*. *frigidus*, *Thienemanniella* sp., and *Chaetocladius* sp., belong to Chironomidae. This is in accordance with the ecology of these taxa, as they are all three typical inhabitants of fast-flowing waters [44]. *Scatella tenuicosta* as indicator for rheocrene springs is somewhat controversial, as the species is clearly linked to hot springs. Seven out of the ten hot springs examined in this study were rheocrene, which might result in a bias in the dataset. Indicator species for limnocrene springs were the chironomids *C*. *tibialis* and *Arctopelopia* sp., which have both mainly been reported from lentic or slow-flowing waters in Iceland [44], as well as Cladocera.

In our study, altitude was not related to diversity nor community composition, even though there were some clear trends related to altitude. The chironomids *Chaetocladius* spp., *Limnophyes* sp, and *Metriocnemus* spp. (all Orthocladiinae) were for example mainly found in highland springs (above 300 m asl), and *Parochlus kiefferi* Garrett (Podonominae) was exclusively found in highland springs in this study. This distribution pattern could be due to additional characteristics of highland springs other than high altitudes. Springs in the Central Highlands of Iceland are often isolated, both from other water bodies and from human activity (e.g., traffic, agriculture, and urban areas). Highland springs are thus less exposed to disturbance, which might be reflected in a more intact moss cover surrounding the spring, providing habitat for spring invertebrates [45–47]. The chironomid taxa predominantly found in highland springs, *Chaetocladius* spp., *Limnophyes* sp, and *Metriocnemus* spp., are all semi-aquatic and thrive on emerging mosses in the transition zone between terrestrial and freshwater habitats. *P*. *kiefferi* is likewise reported from mosses in springs [44,46].

Community composition at the source was linked to geographical position, namely latitude, of the spring. A molecular study on the endemic groundwater amphipod *C*. *islandicus* found in Icelandic springs [48] showed that geographical distances between sampling sites were reflected in genetic divergence between monophyletic groups of the species. This provides evidence that geographical position can be an important variable in shaping community composition by determining species distribution, especially in taxonomic groups with slow dispersal abilities (e.g., Crustacea), in comparisons to taxonomic groups with fast dispersal abilities (e.g., insects) that often have flying adult stages. As discussed for altitude, geographical distances between sampling sites are related to the variable isolation from other water bodies, which has not been analysed in this study but should be included in further analyses.

Taxa richness was lower and evenness slightly higher at the source than at the surface. This may indicate that the source community is made up of fewer and more evenly distributed taxa compared to the surface community. This could be because the source is a more “simple” and more stable habitat and seems to be less affected by environmental variables than the surface habitat. Spring type, which determines the habitat structure and hydraulic conditions around the source, is an important variable for the surface community but not for the source community.

Contrary to our prediction (iv), beta diversity was higher at the source than at the surface, indicating that source samples were more variable than surface samples. In cold springs, the taxa composition, consisting mainly of common and ubiquitous species of the Icelandic freshwater fauna, e.g., *O*. *frigidus*, *E*. *minor*, and *Diamesa* spp., indicates that the source community is less specialized and rather opportunistic, and common species exhibiting good dispersal abilities and a broad tolerance-range towards environmental variables such as temperature, pH, or oxygen availability, may have competitive advantage over species with a narrower tolerance range. Chironomids of the genera *Diamesa*, *Orthocladius*, and *Eukiefferiella* were also found to be the initial colonizers in glacial streams in Alaska [2]. The higher beta diversity at the source could be explained by a stochastic colonization of the source habitat by invertebrates from the adjacent surface habitat, and the low number of stygobiont species found, namely only the amphipod *Crangonyx islandicus*. A high site-to-site variation may indicate that the community assembly is dominated by stochastic rather than deterministic processes, as stated in prediction (iii). Chase [1] proposed that the relative importance of deterministic assembly processes increases under “harsh” environmental conditions, e.g., high disturbance, low productivity, or predation pressure, resulting in a lower beta diversity [1,4]. The spring source is characterized by more stable conditions than further downstream, and could thus be considered a less harsh environment. Stochastic processes might dominate the community assembly at the source and lead to a dispersal-assembled community. Which species of a broadly tolerant species pool come first to colonize a spring source might be more a matter of chance than of niche adaptation, as reflected in the higher within-sites variation of source samples. It has been suggested that insects found in springs are not necessarily stenobiontic but instead represent parts of populations with a more flexible ecology [35]. These ecologically flexible individuals might be attracted to spring habitats due to ease of oviposition. A colonization of spring sources by broadly tolerant species rather than specialists indicates again that the source community of cold springs is dispersal-assembled. The source community of hot springs seems more likely to be niche-assembled. There, species which are adapted to high water temperatures and often high concentration of ions can successfully colonize (e.g., [49]). This supports the hypothesis that strong ecological filters (“harsh” environments) favour niche-assembly [1].

Although it would have been preferred to employ the same sampling methods at both types of habitats within each spring, we were restricted by the physical structure of each habitat. Collecting Surber samples within the source was impossible because of the dimensions, and pilot work revealed that physical scrubbing was too destructive to the source habitat. On the other hand, electrobugging on the benthic surface would be highly dependent on water current, which differed among springs, especially between limnocrene and rheocrene springs, to carry invertebrates into the net.

The lower taxa richness observed at the source than in the surface samples could be explained by having Crustacea only partially identified to species level. Many Crustacean species are known to be creno- or stygobiontic [50], which could underestimate the diversity at the source as opposed to the surface. However, the known Crustacean fauna of Icelandic springs consists–with the exception of two groundwater amphipods–mostly of ubiquitous aquatic taxa than obligate groundwater dwellers, although some Ostracoda (e.g., *Potamocypris pallida*) might be crenobiontic in Iceland [51]. This absence of an extensive stygobiontic and crenobiontic fauna as compared to other parts of the world leads us to the conclusion that the patterns observed in our data are unlikely to only be artefacts of sampling methods.

It has been proposed that species diversity is generally low in springs and spring brooks due to their temperature stability which consequently reduces thermal niches and potentially taxa richness [49,52]. Thus, springs might have a low alpha diversity, but beta diversity on the other hand might be high and contribute greatly to the overall freshwater diversity of a region [49]. Taxa richness of the springs in this study varied between 1 and 22 taxa (on average 11) in the surface samples, and between 1 and 18 taxa (on average 8) in the source samples. Those numbers are on average indeed lower than invertebrate taxa richness found in studies in other water bodies in Iceland [53]. However, the total number of invertebrate taxa found in our study (gamma diversity) was 54, which is comparable to the overall number of invertebrate taxa reported from Icelandic rivers, e.g., 52 invertebrate species from the river Laxá [54].

## Conclusion

The main drivers of invertebrate community composition in Icelandic springs were water temperature, spring type, and geographical position (latitude). The assembly mechanism of the source communities appears to depend on water temperature, with hot springs being more niche-assembled, whereas cold springs are more dispersal-assembled. Although the environmental variables at the source and the surface location were similar within a spring, the source community was more influenced by groundwater as seen for example in the occurrence of groundwater amphipods. This resulted in different patterns of diversity between source and surface; the source communities were more variable between springs, whereas surface communities differed according to spring type. This emphasises the need for a clear terminology in spring research, as the exact location of sampling can have implications on the data obtained and thus the conclusions drawn.

Due to their temporal stability in chemical and physical variables, springs may act as a refugia for freshwater organisms in rapidly changing environments, and an understanding of the processes governing community structure is thus becoming increasingly important. Although Icelandic freshwaters are relatively species-poor, their natural temperature gradients make them useful to test theoretical predictions. Despite individual springs having a low species diversity, a large portion of the Icelandic freshwater fauna is represented in springs from different locations and with different ecological characteristics, and we argue that more emphasis should be put on keeping spring habitats intact on a global scale.

## Supporting information

S1 Table Alpha diversity indices of all sites in a study on invertebrate diversity in Icelandic springs. Two locations were sampled in each spring, source and surface. Mean taxa richness (± standard deviation) was 8 ± 3.8 at the source and 11 ± 4.3 at the surface, mean Shannon diversity 3.5 ± 1.48 and 4.0 ± 2.00, and mean evenness 0.5 ± 0.27 and 0.4 ± 0.21, respectively.
(DOCX)
