# Supplementary material for: Invertebrate communities in springs across a gradient in thermal regimes
Source: PLoS One. 2022 May 5;17(5):e0264501. doi: 10.1371/journal.pone.0264501 (PMC9070909; doi:10.1371/journal.pone.0264501)
Supplement: S1 Table — Two locations were sampled in each spring, source and surface. Mean taxa richness (± standard deviation) was 8 ± 3.8 at the source and 11 ± 4.3 at the surface, mean Shannon diversity 3.5 ± 1.48 and 4.0 ± 2.00, and mean evenness 0.5 ± 0.27 and 0.4 ± 0.21, respectively. (DOCX) [file pone.0264501.s001.docx]

**S1 Table.** **Alpha diversity indices of all sites in a study on invertebrate diversity in Icelandic springs.** Two locations were sampled in each spring, source and surface. Mean taxa richness (± standard deviation) was 8 ± 3.8 at the source and 11 ± 4.3 at the surface, mean Shannon diversity 3.5 ± 1.48 and 4.0 ± 2.00, and mean evenness 0.5 ± 0.27 and 0.4 ± 0.21, respectively.

| **Spring** | **Source** | | | **Surface** | | |
| --- | --- | --- | --- | --- | --- | --- |
|  | Taxa richness | Shannon diversity | Evenness | Taxa richness | Shannon diversity | Evenness |
| Botnar I | 3 | 2.71 | 0.91 | 10 | 3.69 | 0.37 |
| Botnar II | 13 | 5.59 | 0.43 | 18 | 1.66 | 0.09 |
| Dynjandi | 10 | 2.27 | 0.23 | 16 | 4.92 | 0.31 |
| Enni | 17 | 1.74 | 0.10 | 16 | 4.24 | 0.27 |
| Friðsæld | 9 | 3.36 | 0.37 | 11 | 2.83 | 0.26 |
| Gæsavötn | 6 | 4.26 | 0.71 | 20 | 7.32 | 0.37 |
| Galtalækur | 7 | 3.41 | 0.47 | 12 | 6.58 | 0.55 |
| Goðdalafjall | 12 | 3.95 | 0.33 | 13 | 4.44 | 0.34 |
| Grænavatn Norður | 4 | 3.57 | 0.89 | 13 | 4.45 | 0.34 |
| Grænavatn Suður | 6 | 4.15 | 0.69 | 6 | 3.70 | 0.62 |
| Hænuvík | 14 | 2.30 | 0.16 | 12 | 1.72 | 0.14 |
| Hafgrimsstaðir | 8 | 6.36 | 0.79 | 7 | 2.08 | 0.30 |
| Hagalækur | 7 | 2.14 | 0.31 | 8 | 3.36 | 0.42 |
| Háöldur | 10 | 6.02 | 0.60 | 10 | 2.61 | 0.26 |
| Hengill IS6a | 7 | 2.05 | 0.29 | 14 | 1.45 | 0.10 |
| Hengill IS7 | 2 | 1.89 | 0.94 | 9 | 5.53 | 0.61 |
| Hengill IS8 | 18 | 4.18 | 0.23 | 12 | 3.47 | 0.29 |
| Herðubreiðarlindir | 12 | 5.99 | 0.50 | 14 | 3.79 | 0.27 |
| Hofsvellir | 8 | 6.75 | 0.84 | 15 | 4.00 | 0.27 |
| Hörgshlíð | 6 | 5.50 | 0.92 | 7 | 2.09 | 0.30 |
| Hraun | 11 | 5.97 | 0.54 | 13 | 3.70 | 0.28 |
| Hrauná | 13 | 3.41 | 0.26 | 22 | 9.02 | 0.41 |
| Hruni | 8 | 2.24 | 0.28 | 10 | 3.57 | 0.36 |
| Kálfaströnd | 5 | 4.59 | 0.92 | 7 | 5.10 | 0.73 |
| Kiðárbotnar | 7 | 2.71 | 0.39 | 8 | 5.23 | 0.65 |
| Klapparós | 3 | 2.87 | 0.96 | 10 | 3.14 | 0.31 |
| Krákárbotnar | 8 | 2.59 | 0.32 | 10 | 3.20 | 0.32 |
| Lækjarbotnar Hol | 7 | 2.91 | 0.42 | 11 | 2.58 | 0.23 |
| Lækjarbotnar Rvk | 1 | 1 | 1 | 8 | 2.08 | 0.26 |
| Langivogur | 6 | 4.36 | 0.73 | 7 | 4.04 | 0.58 |
| Lón | 5 | 3.65 | 0.73 | 2 | 1.65 | 0.82 |
| Mælifellslaug | 11 | 2.09 | 0.19 | 13 | 3.27 | 0.25 |
| Miðhúsaskógur | 12 | 3.16 | 0.26 | 10 | 3.26 | 0.33 |
| Mótunga | 6 | 4.71 | 0.79 | 15 | 5.23 | 0.35 |
| Nauteyri | 12 | 4.01 | 0.33 | 12 | 2.40 | 0.20 |
| Oddar | 10 | 2.61 | 0.26 | 8 | 3.35 | 0.42 |
| Presthólar | 10 | 1.66 | 0.17 | 6 | 4.36 | 0.73 |
| Sandur | 7 | 3.81 | 0.55 | 8 | 6.40 | 0.80 |
| Sikið | 1 | 1 | 1 | 14 | 2.30 | 0.16 |
| Sílatjörn | 9 | 2.40 | 0.27 | 12 | 5.38 | 0.45 |
| Skarðslækur | 11 | 4.22 | 0.38 | 14 | 8.26 | 0.59 |
| Staðarhraun Bær 1 | 8 | 3.24 | 0.40 | 15 | 3.29 | 0.22 |
| Staðarhraun Kirkja | 8 | 2.38 | 0.30 | 11 | 3.65 | 0.33 |
| Steinsstaðir | 4 | 2.56 | 0.64 | 5 | 1.37 | 0.27 |
| Svartárbotnar | 6 | 2.48 | 0.41 | 12 | 5.22 | 0.44 |
| Svartárkot | 2 | 1.89 | 0.94 | 8 | 3.50 | 0.44 |
| Þverá | 8 | 3.64 | 0.46 | 18 | 10.90 | 0.60 |
| Úlfsstaðir | 7 | 6.45 | 0.92 | 1 | 1 | 1 |
| Vatnsvik | 5 | 2.23 | 0.45 | 5 | 4.05 | 0.81 |
